# Porous Mortars Incorporating Active Biochar from Olive Stone Waste and Recycled Masonry Aggregate: Effects of Accelerated Carbonation Curing

**DOI:** 10.3390/ma18040904

**Published:** 2025-02-19

**Authors:** Antonio Manuel Merino-Lechuga, Ágata González-Caro, Álvaro Caballero, José Ramón Jiménez, José María Fernández-Rodrígez, David Suescum-Morales

**Affiliations:** 1 Área de Ingeniería de la Construcción, E.P.S de Belmez, Universidad de Córdoba, 14240 Córdoba, Spain; ammlechuga@uco.es (A.M.M.-L.); p02sumod@uco.es (D.S.-M.); 2 Área de Química Inorgánica, E.P.S de Belmez, Universidad de Córdoba, 14240 Córdoba, Spain; q32gocaa@uco.es (Á.G.-C.); alvaro.caballero@uco.es (Á.C.); 3 Instituto Químico para la Energía y el Medioambiente (IQUEMA), Universidad de Cordoba, 14071 Cordoba, Spain

**Keywords:** construction and demolition waste, heat of hydration, CO_2_ capture, accelerated carbonation curing, sustainable construction materials, olive stone waste, carbon sequestration

## Abstract

This study investigated the use of activated biochar derived from olive stone waste and recycled masonry aggregates in porous mortar mixtures and assessed their behaviour under accelerated carbonation curing conditions. Three mortar mixtures were produced, incorporating 0%, 5%, and 10% activated biochar by volume. The physical, chemical, and mechanical properties of the mortars were analysed, including the compressive strength, flexural strength, water absorption, porosity, and CO_2_ capture capacity. Additionally, calorimetry tests were performed on cement pastes with 0%, 0.5%, 1%, 3%, 15%, and 20% activated biochar to evaluate their impact on setting times and ensure compatibility between activated biochar and cement. The results showed that the addition of biochar improved mechanical properties, particularly under accelerated carbonation curing, whereas active biochar (AcB) significantly enhanced the compressive and flexural strengths. Furthermore, biochar incorporation boosted CO_2_ capture efficiency, with the 10% biochar mix showing up to 147% higher CO_2_ uptake, compared with a control. These findings suggest that activated biochar and recycled masonry aggregates can be effectively utilised to develop sustainable construction materials and thereby contribute to carbon sequestration and the reduction in environmental impacts. This research fills the gaps in the current knowledge on the use of activated biochar from olive stones waste in cement-base materials under accelerated carbonation conditions.

## 1. Introduction

The construction industry is a major contributor to global waste generation, producing over three billion tons of construction and demolition waste (CDW) annually and accounting for approximately 36% of the world’s total waste output [1,2,3,4,5]. In the United States, CDW increased from 50 million tons in 1980 to 600 million tons in 2018 [5,6], and projections estimate the annual CDW generation between 2022 and 2026 to be 330 million tons [7]. In China, CDW production exceeds 1.5 billion tons annually [5,8,9], with 45.1% of the total attributed to demolition waste [7]. Moreover, the European Union (EU) produces approximately 850 million tons of CDW per year, accounting for 31% of its total waste [5]. Despite the high recycling rate of CDW in the EU (89%), much of it remains of low quality [10]. China aims to increase CDW utilisation to 60% by 2025 [11], and research suggests that up to 80% of CDW can be repurposed as recycled materials [7,11].

A promising application of CDW is the production of recycled aggregates (RA) as replacements for natural aggregates (NA) in concrete, which can help mitigate both resource scarcity and waste accumulation [12]. RA is produced by crushing, washing, and screening materials, such as waste concrete and bricks; however, [13] the adhered mortar (AM) present in RA can weaken the interfacial transition zone (ITZ), which potentially leads to microcracking under harsh conditions [7,14,15,16]. Nevertheless, the use of recycled concrete aggregates (RCA) and recycled mixed aggregates (RMA) in concrete has shown favourable effects on the physical-mechanical properties, durability, CO_2_ absorption, and overall lifecycle sustainability [17,18,19,20,21,22,23,24]. Although RCA and RMA usage of up to 30% as NA replacement shows minimal negative impact on concrete properties [25,26], the fine fraction of CDW, which is known as construction and demolition waste powder (CDWP), is often neglected.

CO_2_ is widely recognised as a leading greenhouse gas driving global warming [27]. Emerging economies rich in natural resources and seeking technological advancements, face the challenge of promoting economic growth while ensuring environmental sustainability [28,29]. The construction sector is a major contributor to CO_2_ emissions and therefore must be a focal point for mitigation strategies [30,31,32,33,34]. Accelerated carbonation curing, or CO_2_ curing, has shown promise in enhancing the early mechanical strength while sequestering large amounts of CO_2_ over a short period [35,36]. This technique is particularly relevant in the precast concrete industry, in which early strength is crucial for increased productivity [37,38,39,40].

The global agriculture and food processing sectors generate approximately 140 billion tons of biomass waste annually [41,42,43]. Activated carbons are commonly produced from carbon-rich raw materials, such as coal; however, biomass, such as fruit stones and nut shells, are increasingly used [44,45,46,47,48,49]. Biochar has attracted attention as an alternative adsorbent for gases and organic compounds [44,46,50,51,52,53,54,55,56,57]. Olive stone waste, which comprises approximately 9–27% of processed olives, can be used as a precursor for activated carbon and has proven highly effective in CO_2_ capture [56,58,59,60,61,62,63,64,65,66,67,68,69,70,71,72,73]. Although few studies have focused on the use of olive stone-derived activated carbon for carbon sequestration, further research could help develop sustainable carbon capture methods.

Recent studies have explored the incorporation of activated carbon into construction materials; however, such research is still limited [74,75,76,77,78,79,80,81]. Although biochar has been extensively studied [82,83,84,85,86,87,88,89,90,91,92,93], there is a lack of investigations into the effects of olive stone-derived activated carbon on mortars and concrete [94,95]. Some studies have indicated improvements in mechanical properties with certain proportions of biochar or activated carbon [76,80,82,83,85,89,94,96,97]. However, no studies have examined the use of olive stone-derived activated carbon in porous mortars under accelerated carbonation conditions (CO_2_ curing). This study aims to fill this gap by investigating the environmental benefits and mechanical performance of mortars that incorporate olive stone-derived activated carbon, which can act as a long-term carbon sink. In addition, this study introduces an ultrafast test method that uses CO_2_ isotherms to assess the CO_2_ adsorption capacities of these materials.

While research on waste-derived additives in cementitious systems has grown, there remains a notable gap in understanding how olive stone-derived activated carbon performs under accelerated carbonation conditions.

This study addresses two key objectives: first, to evaluate whether incorporating activated biochar at concentrations of 0%, 5%, and 10% enhances the CO_2_ capture capacity of porous mortars without compromising mechanical performance, and second, to determine whether this bio-based additive alters the setting times of cement paste. The underlying hypothesis is that olive stone-derived activated carbon can effectively increase CO_2_ capture without significantly affecting setting times or mechanical properties, indicating good compatibility within the cement matrix. Accordingly, the present work provides a thorough examination of the environmental benefits (CO_2_ capture) and mechanical behaviour of mortars containing different dosages (0%, 5%, and 10%) of activated carbon. In addition, the heat of hydration of cement pastes with varying proportions of activated biochar (0%, 0.5%, 1%, 3%, 15% and 20%) was assessed to ascertain its impact on setting times, with no substantial variations observed. By using advanced characterisation techniques, including the Brunauer–Emmett–Teller (BET) adsorption method, X-ray diffraction (XRD), heat of hydration, thermogravimetric analysis (TGA), scanning electron microscopy (SEM), and transmission electron microscopy (TEM), alongside a rapid CO_2_ adsorption test, this study not only clarifies the influence of this bio-based additive on the physical and mechanical properties of porous mortars but also offers novel insights into the development of more sustainable construction materials.

## 2. Materials and Methods

### 2.1. Materials

For this study, recycled masonry aggregate (RMA) was used, which had been previously employed in several studies by the authors [35,98,99]. The RMA was composed of approximately 53% red ceramic brick, 39.8% masonry mortar, 5.7% unbound aggregates, 0.4% concrete, and 0.2% gypsum particles. Additionally, activated carbon (produced by steam activation) was derived from olive stones from the bioenergy plant of S.C.O. El Tejar (Córdoba, Spain). The details of their synthesis and treatment processes can be found in the references [60,100,101,102]. AcB was ground in a steel ball mill for 2 h to reduce its grain size as much as possible. The cement used in this study was CEM II/A-L 45.2 R (UNE-EN 197-1:2011) [103], The cement was manufactured by Votorantim Cementos, Córdoba, Spain.

Table 1 presents the main physical–mechanical properties of the RMA: the dry bulk density (UNE-EN 1097-6:2014 [104]), water absorption (UNE-EN 1097-6:2014 [104]), and sand equivalent (UNE-EN 933-8:2012 + A1/1M:2016 [105]).

### 2.2. Mix Design

The goal was to create a dry porous mix (PM) comparable to that produced in precast concrete plants using only fine materials [35,38,39,40]. To achieve this, the particle size distribution of the aggregate (RMA) was adjusted to satisfy the lower limit of ASTM C 144-04 [106]. The RMA was sieved through various sieve sizes, as listed in Table 2, and the 0.25/0.5 mm and <0.125 mm fractions were removed. Table 2 lists the quantities of RMA used (kg/m^3^) based on the results of previous studies [35,97,107] and the adjustments. The volume of added cement was determined using a 1/6 (cement/aggregate) ratio. Given the different densities of cement and active biochar (AcB) (2.89 versus 1.73 g/cm^3^, respectively), additions were made by volume. Tap water was used, resulting in three different mixes that varied only in the amount of AcB added: 0%, 5%, and 10%, named PM-0, PM-5, and PM-10, respectively. Table 2 lists all quantities used.

The kneading process was as follows: RMA and saturated water were combined and mixed for 90 s [108]. After a 5 min interval, cement and AcB were introduced and mixed for 30 s. Subsequently, kneading water was added and mixed for another 90 s. The resulting mixtures had a dry consistency, necessitating ‘vibro-compaction’ similar to the methods used in precast concrete plants [35,38,39,40,109]. This ‘vibro-compaction’ method has also been employed in other studies [97,107]. Samples were formed in 4 cm × 4 cm × 16 cm moulds using a specialised compactor and the Harvard miniature compaction technique (10 strokes per 2 layers). After 3 h, the samples were removed from the moulds.

After demoulding, the specimens were cured in parallel for 1, 3, and 7 days in two distinct environments to enable direct performance comparisons under normal and accelerated carbonation conditions. In the Normal Climatic Chamber (NCC), the temperature was maintained at 21 ± 2 °C, with 65% relative humidity (RH) and a CO_2_ concentration of approximately 0.04% (typical atmospheric level). In contrast, the Accelerated Carbonation Chamber (ACC) replicated the same temperature and RH conditions but increased the CO_2_ concentration to 5% using a Climacell 707-Evo MMM device, MMM Group, Munich, Germany. The CO_2_ supply was continuously regulated using an automated control system that monitored and adjusted the gas flow to maintain a stable 5% concentration. To ensure homogeneous exposure, the specimens were evenly spaced within the chamber, and air circulation was optimised to prevent CO_2_ stratification. The curing chamber was periodically checked for deviations in temperature, humidity, and CO_2_ concentration to ensure consistent test conditions across all specimens.

### 2.3. Characterisation and Testing Methods

The chemical compositions of the raw materials (cement, AcB, and RMA) were analysed via X-ray fluorescence (XRF) using a ZSX Primus IV (Rigaku, Tokyo, Japan). Additionally, X-ray diffraction (XRD) was performed on both raw and hardened samples using a Bruker D8 Discover A25 (Billerica, MA, USA), and thermogravimetric (TGA) and differential thermal analyses (DTA) were conducted using a Setaram Setsys Evolution 16/18 (Setaram, Freiberg, Germany) at a heating rate of 5 °C/min up to 1000 °C. The particle size distributions of the cement and AcB were measured using a Mastersizer S (Malvern Instruments, Malvern, UK) with ethanol as the dispersant.

Transmission electron microscopy (TEM) with energy dispersive spectroscopy (EDS) was employed to examine the microstructure of AcB using a Talos F200i (Thermo Fisher Scientific, Waltham, MA, USA). The CO_2_ capture capacity of AcB was determined at 30 °C with a PCTPro-2000 volumetric analyser (Merck, Darmstadt, Germany), following a degassing process at 40 °C. CO_2_ isotherms were studied from 0 to 39 atm in increments of 3.5 atm. The results were verified via triplicate measurements.

The heat of hydration of cement mixtures with activated biochar (AcB) was measured using an 8-channel TAM air conduction calorimeter (TA Instruments, New Castle, DE, USA) at a controlled temperature of 25 °C. To assess how the heat of hydration and the setting time of the cement paste varied with AcB addition, mixtures were prepared with 0%, 0.5%, 1%, 3%, 15%, and 20% AcB. Data were collected for up to 10,000 min (approximately 166.7 h) from the onset of the hydration of the paste.

The flexural and compressive strengths of the prismatic samples were tested at 1, 3, and 7 d, according to EN 1015-11:2019. For the quantification of flexural strength, three specimens of each mixture were evaluated using prismatic samples with the sizes of 40 mm × 40 mm × 160 mm, while six semi-prismatic samples with the sizes of 40 mm × 40 mm × ≈80 mm were used for the compressive strength. Dry bulk density, water absorption, and accessible porosity were determined at 7 d according to UNE 83980, with three repetitions performed for each mixture. The sample (AcB) was analysed using scanning electron microscopy (SEM) with a JEOL 7800 (Tokyo, Japan), where a gold sputter coating improved the image quality. The carbonation depths of the hardened samples were assessed using a phenolphthalein spray prepared in a solution of deionised water and ethyl alcohol.

**Figure 1:**
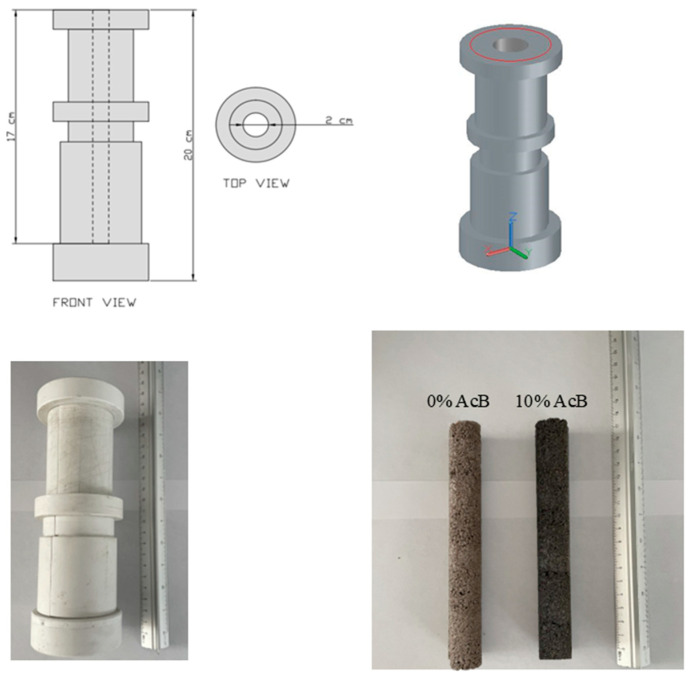
Custom wooden mould and tested PM-0 and PM-10 samples.

The CO_2_ capture capacities (0 to 35 atm) for the PM-0 and PM-10 samples were analysed using cylindrical specimens (17 cm in height, 2 cm in diameter) at 30 °C. The pressure was limited to 35 atm to avoid damaging the samples, as opposed to the 39 atm pressure used for AcB. This analysis was performed using PCTPro-2000. The mould was made of wood, and Figure 1 shows the test samples. Degassing was carried out at 40 °C for 1 h under standard vacuum conditions to prevent gas contamination in the equipment, without using a turbomolecular pump. TGA and DTA were also conducted at 1, 3, and 7 d on hardened mortar samples under both curing environments.

## 3. Characterisation of Raw Materials

Table 3 shows the chemical compositions of the RMA, cement, and AcB that were analysed using XRD. The results for the RMA and cement are consistent with the findings of previous studies [35,110,111]. Notably, a high carbon content was observed in the activated carbon, reflecting its CO_2_ balance (and other elements with atomic numbers equal to or lower than that of oxygen). Additionally, trace amounts of elements, such as calcium, chlorides, sulphates, and phosphates, were detected, similar to the composition reported by Irshidat et al. [112].

**Figure 2:**
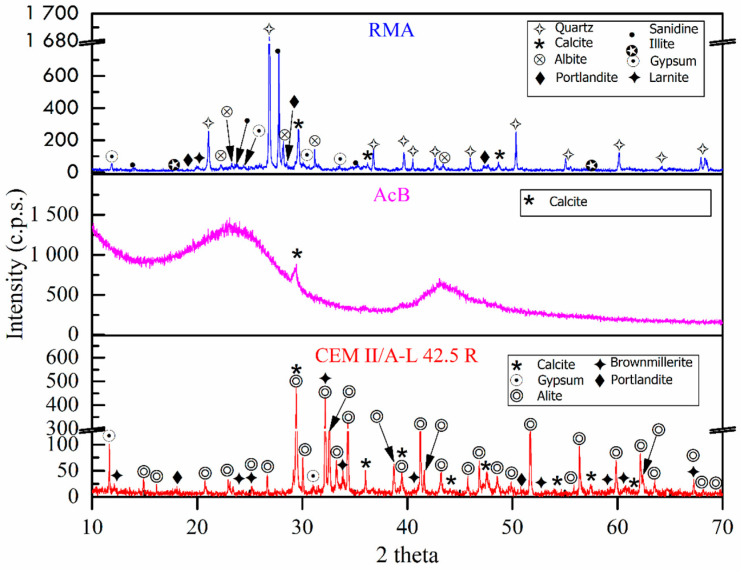
X-ray diffraction of raw materials used.

Figure 2 shows the XRD patterns of the raw materials. For AcB, two broad peaks were found at a 2θ value of 24° and 43°, which would correspond to the (002) and (100) crystalline planes of the graphite structure [54,61]. Moreover, a peak was found at the 2θ value of 31°, corresponding to calcite (CaCO_3_) (05-0586). These results agree with the XRF results that indicated that CaO was the main oxide detected. These results support the previously mentioned XRF results. The phases found for the cement and RMA were the same as those identified in previous studies [110,111,113,114] and were similar to those found by Saiz-Martínez et al. [114] for recycled aggregates from bricks, and by Reig et al. [115] in ceramic waste.

**Figure 3:**
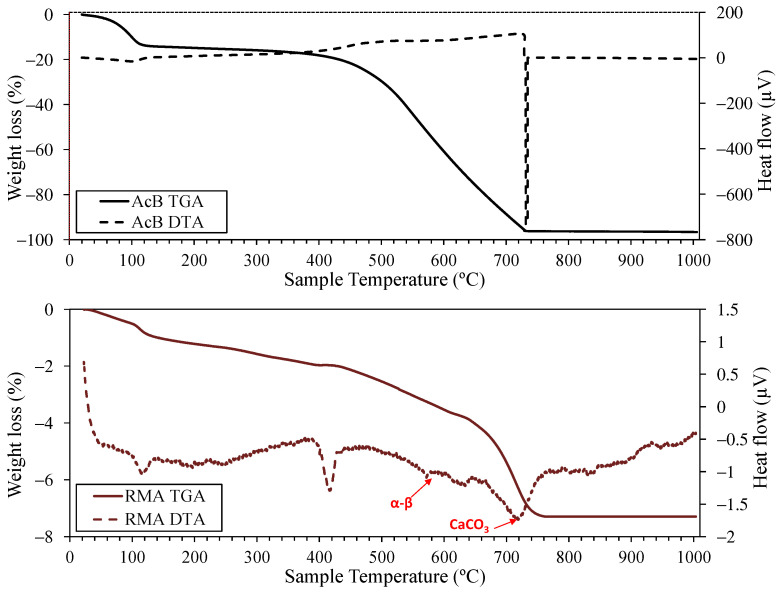
TGA and DTA of active biochar (AcB) and recycled masonry aggregate (RMA).

Figure 3 presents the thermogravimetric results for AcB and RMA. For AcB, a weight loss of 3.6% was noted between 110 and 400 °C, likely owing to sulphur evaporation [60]. A significant weight loss of 79% occurred from 400 to 720 °C, with two exothermic peaks detected from 460 to 480 °C and at 720 °C. These can be linked to the breakdown of the remaining organic matter [116]. A final residue of 3.3% remained, indicating the high purity of the activated carbon [101,117].

Five distinct phases were observed in the RMA. The first, from room temperature to 110 °C, shows a 0.56% weight loss owing to moisture, which is consistent with the findings of Gonzalez-Corominas et al. [118]. The second stage (110–380 °C) includes a peak at 130 °C that is attributable to water loss from the hemihydrate [119] with dehydration of calcium silicates and aluminates [120]. The third stage (380–480 °C) marks portlandite calcination at approximately 410 °C [118,121]. Between 480 and 640 °C (fourth stage), carbonates formed during hardening decomposition, and quartz transformed from the α to the β phase at 570 °C [122]. These are clearly identifiable owing to the quartz content of the RMA. The fifth stage (640–1000 °C) involves calcium carbonate breakdown [123], although some researchers, including Sáez del Bosque et al. [124], merged the fourth and fifth stages into a single section. Within this 480–1000 °C range, peaks were identified at 560–564 °C, 700–748 °C, and 860–879 °C. Considering the dry bulk density of the RMA (Table 1) and the 4.93% weight loss owing to calcium carbonate calcination, the calcium carbonate content of RMA is estimated to be 239 kg/m^3^. Further detailed information on the material characteristics is provided by the authors of other studies [35].

**Figure 4:**
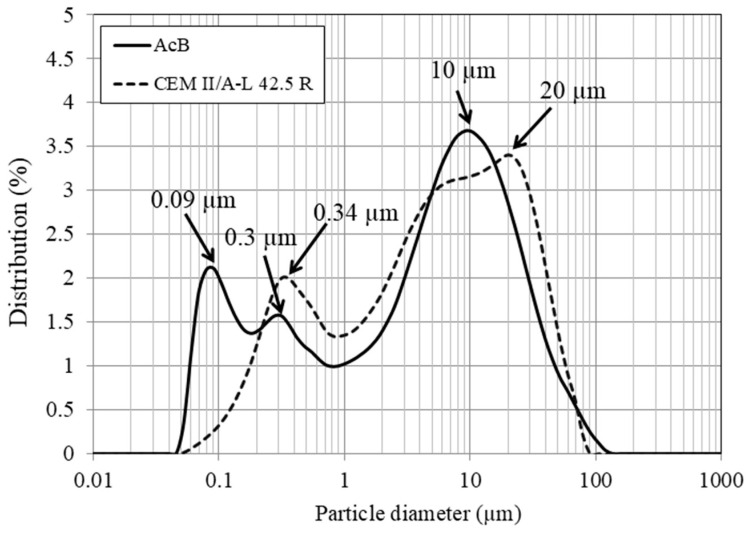
Particle sizes of active biochar (AcB) and cement.

Figure 4 presents the particle size distribution of cement and AcB, both of which range from 0.05 to 100 µm. The cement exhibits a trimodal distribution similar to that of AcB. The majority of particles measured approximately 10 µm for AcB and 20 µm for cement.

**Figure 5:**
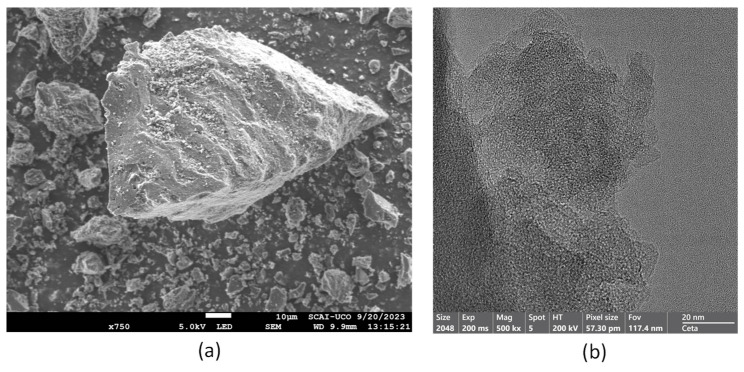
SEM (**a**) and TEM (**b**) images of active biochar (AcB).

The morphology of AcB was examined using SEM and TEM (Figure 5a,b), respectively. AcB exhibited a highly porous structure with large cavities and irregular angular shapes [112,125]. Most particles were approximately 10 µm in size, consistent with the particle size distribution shown in Figure 4. SEM analysis revealed numerous pores of both micron and submicron sizes [64,68], which enhanced adsorption as larger pores channelled substances into smaller pores [61,73]. The results of a TEM analysis confirmed the presence of surface voids, which increased the contact area between the adsorbent and adsorbate, which is ideal for adsorption [69,81]. The darker areas represent the biochar, while the lighter areas correspond to the pores.

**Figure 6:**
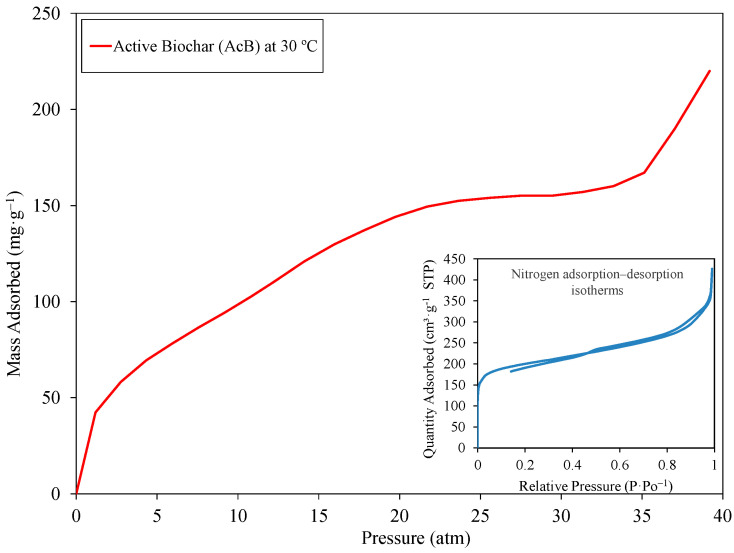
CO_2_ adsorption equilibrium isotherms and nitrogen absorption-desorption isotherms of active biochar (AcB).

The CO_2_ adsorption performance of AcB was assessed using equilibrium isotherms (Figure 6), which showed an increase in adsorption with increasing pressure. The isotherm resembled a type IV isotherm, as classified by the IUPAC [126,127]. The sample achieved its maximum adsorption capacity at 39 atm and 30 °C with a value of 220.35 mg·g^−1^. These results agree with the morphology observed via SEM and TEM, which reveals a porous structure with voids [61,69,73]. J. Serafin et al. [71] reported a CO_2_ capture capacity of 6.32 mmol·g^−1^ (278.08 mg·g^−1^) at 0 °C under ambient pressure, which is higher than the results of the current study. This higher capacity may be attributed to the larger SBET of 915 m^2^·g^−1^ achieved by those authors. In contrast, Puig-Gamero et al. [58] found a lower adsorption capacity of 4.66 mmol·g^−1^ (205.04 mg·g^−1^) at 20 bar and 0 °C. Under similar conditions, our study yielded a lower adsorption capacity of approximately 146 mg·g^−1^. The reduced capacity observed here could be explained by the difference in BET surface area, as the previous study [58] reported 1190 m^2^·g^−1^, whereas the AcB used in our work had a surface area of 632.79 m^2^·g^−1^. These variations in CO_2_ capture performance across studies emphasise the influences of factors such as SBET, pressure, and temperature.

## 4. Results and Discussion

### 4.1. Physico-Mechanical Testing

#### 4.1.1. Compressive and Flexural Strengths

**Figure 7:**
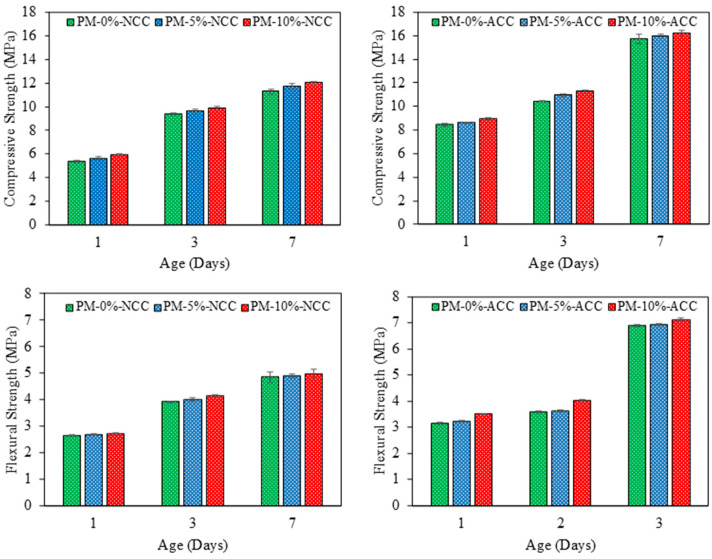
Compressive and flexural strength.

Figure 7 shows the compressive and flexural strength results for various curing periods, percentages of AcB incorporation, and curing environments (NCC and ACC). Under both conditions, increasing the percentage of AcB consistently led to enhanced strength. Under a normal curing environment (NCC), the incorporation of 10% AcB resulted in compressive strength improvements of 110%, 105%, and 106% at 1, 3, and 7 d, respectively.

A study by Chin et al. [76] noted that incorporating activated carbon as a coarse aggregate in lightweight concrete improved its strength owing to the rough, irregular surface of the activated carbon, which enhanced the bond at the cement–paste interface (i.e., the bond between the biochar and cement matrix). Another contributing factor could be the role of activated carbon as a network of microdeposits that release water during the cement hydration process [75]. This mechanism promotes the formation of hydration products, such as C-S-H, which helps reduce the porosity and increase the sample density [84,87,93,94]. In addition, the high porosity of AcB (Figure 5) may lower the ‘effective’ water/cement ratio, sometimes referred to as the local water/cement ratio, which further strengthens the material [97]. Sisman et al. [83] and T. Chen et al. [85] supported these findings by attributing the strength gains to the pore-filling effect, which refines the microstructure. For example, Javed et al. [80] observed that biochar, when substituted for cement, enhances the strength owing to its microfiller effect and internal curing capacity.

The flexural strength under NCC exhibited a similar trend to the compressive strength. The results were noteworthy, especially those for early curing times (e.g., 1 d). These results can be explained by the AcB fibres penetrating the cement paste and providing increased particle cohesion [74,75,76].

The ACC environment yielded better outcomes across all curing periods and AcB percentages. This curing method may prove highly advantageous for unreinforced precast plants by significantly accelerating production and reducing curing time. Notably, the improvement in the compressive strength owing to CO_2_ curing increased as the AcB content increased. For instance, the sample containing 10% AcB demonstrated a 3 d compressive strength of 11.32 MPa under ACC, surpassing the 7 d value of 11.29 MPa under NCC. Studies on the curing of ordinary Portland cement mortar in CO_2_ using olive stone-derived activated carbon are lacking. This behaviour could be attributed to the formation of an interlocking structure with the calcium carbonate generated during carbonation, which contributes to pore densification and refinement [96].

#### 4.1.2. Dry Bulk Density, Water Absorption, and Accessible Porosity

**Figure 8:**
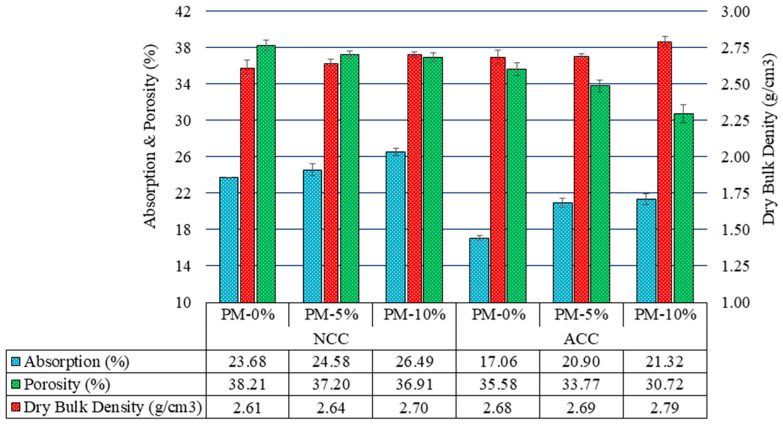
Dry bulk density, water absorption, and accessible porosity at 7 d of curing in both curing environments.

Figure 8 presents the dry bulk density, water absorption, and water-accessible porosity of the samples cured for 7 d in both curing environments. The dry bulk density increased with the rise in AcB content for both environments, aligning with the ‘pore-filler effect ‘described by various researchers [83,89]. These results are attributable to nano-biochar and biochar, and these effects likely account for the observed improvements in mechanical properties with the addition of AcB.

Conversely, the water-accessible porosity (porosity) decreased under both curing conditions. Gupta et al. [82] reported a similar reduction in the capillary water absorption in mortars incorporating biochar. These results suggest that the increase in mechanical strength may be attributable to a lower ‘effective’ water-to-cement ratio [97]. Interestingly, water absorption exhibited an increasing trend with increasing AcB content across both curing environments. These results can be explained by the higher fine-particle content and larger specific surface area of the AcB samples, which allowed them to retain more water.

The influence of the ACC environment was consistent across all the samples, leading to increased dry bulk density, reduced water-accessible porosity, and lower water absorption. These results were largely owing to sample densification caused by carbonation (CaCO_3_ formation) [35,107,128,129]. To date, no studies have investigated the behaviours of samples subjected to accelerated carbonation using biochar derived from olive stones as an additive.

### 4.2. Instrumental Testing

#### Heat of Hydration

**Figure 9:**
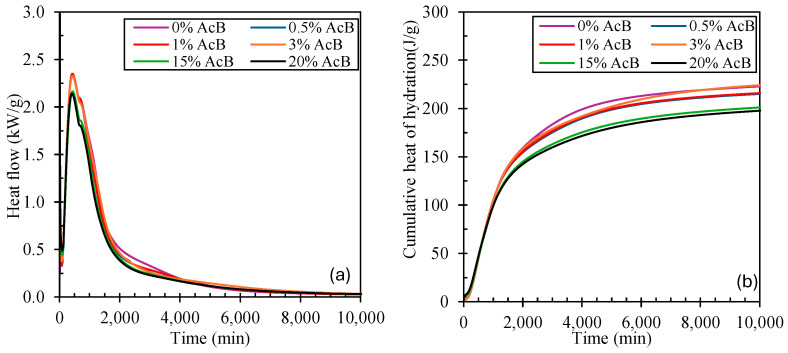
Evolution of the heat flow (**a**) in kW/g and cumulative heat of hydration evolution (**b**) in J/g of cement mixes with additions of 0%, 0.5%, 1%, 3%, 15%, and 20% AcB.

Figure 9a presents the heat evolution curve of the heat of hydration, and Figure 9b illustrates the cumulative heat evolution values for various cement mixtures incorporating AcB.

Regarding the heat of hydration, for all mixtures, irrespective of the amount of AcB incorporated, the initial setting occurred at approximately 300 min. Concerning the heat released, the changes were only evident as a reduction in the heat of hydration with increasing AcB content. These results are consistent with the fact that a lower proportion of cement results in reduced cumulative heat when normalised per gram of sample.

Frías et al. [130] reported a 5 h delay in the setting time when activated carbon was incorporated into the cement paste. Additionally, the delay decreased as the proportion of activated carbon decreased, demonstrating that lower proportions could improve mechanical properties without significantly affecting setting times. Similarly [131], in a study that compared various additives to assess their influence on the setting time and heat of hydration, Bost et al. concluded that cement mixtures incorporating activated carbon exhibited setting times comparable to those observed in this study, with minimal differences compared with the reference samples (cement-only mixtures).

These findings confirm that the activated biochar (AcB) does not interfere with the cement-setting reactions and does not negatively affect its hardening time, making it a material compatible with cement.

### 4.3. X-Ray Diffraction of Mortar

**Figure 10:**
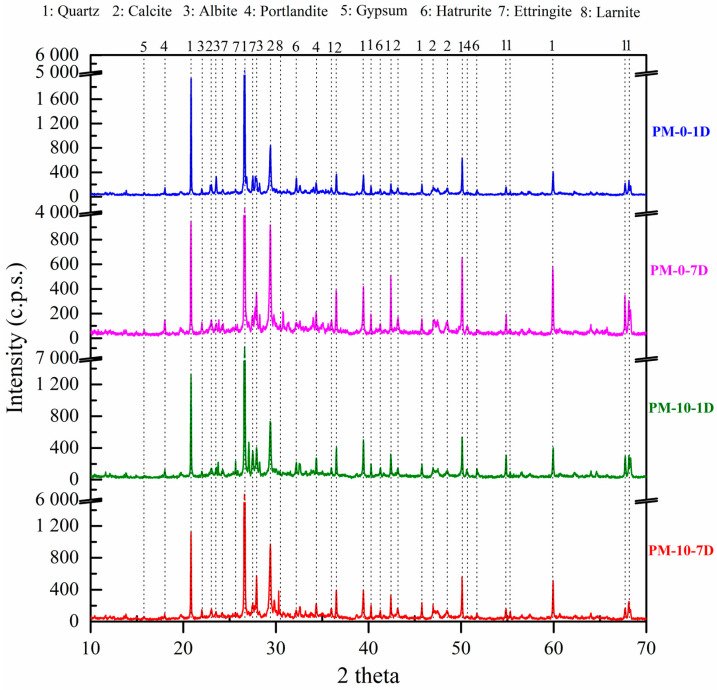
XRD patterns of mortar samples containing 0 and 10% AcB at 1 and 7 d under normal climatic chamber (NCC) conditions.

Figure 10 illustrates the XRD analysis of samples after 1 and 7 d of curing under normal curing conditions (NCC) with 0% and 10% AcB (activated biochar). The identified phases included quartz (05-0490) [132], calcite (05-0586) [132], gypsum (21-0816) [132], and albite (10-0393) [132], all of which were attributed to the sand used. In terms of the hydration products from ordinary Portland cement, the crystalline phases detected were portlandite (44-1481) [132], ettringite (02-0059) [132], hatrurite, and larnite.

A subtle decline in the portlandite intensity was observed at 7 d, which correlated with the increased carbonation depth noted at the same curing time (Figure 10 and Table 4). Notably, no new crystalline peaks appeared with the introduction of AcB, indicating that the olive stone-derived biochar had a minimal impact on the type of hydration products formed. This is consistent with previous research on biochar additives [86,94], where only slight changes in the portlandite intensity were observed when different types of biochar were used [80,87]. These findings were linked to the high specific surface area of the biochar particles, which accelerated hydration and produced more portlandite. However, in our study, this effect was not clearly visible, even though the same trend persisted.

The hatrurite and larnite phases revealed a decrease in the relative intensity peaks at 1 d, which became nearly indiscernible at 3 and 7 d with the addition of 10% AcB. These results suggest that AcB acts as a microdeposit network to facilitate water supply and enhance cement hydration, which likely contribute to the reduced porosity and increased sample density owing to improved C-S-H formation (Figure 8) [75].

**Figure 11:**
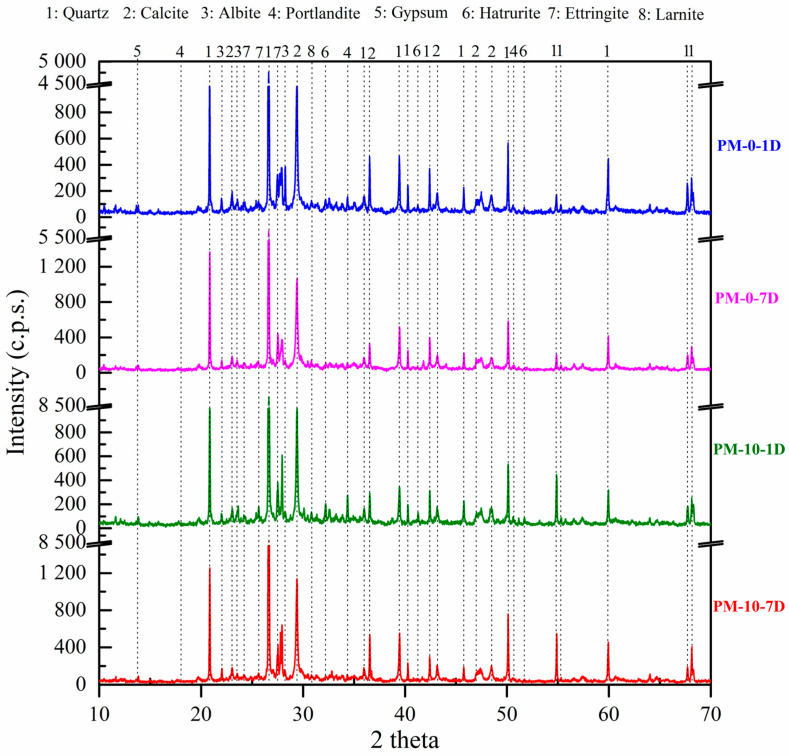
XRD patterns of mortar samples containing 0 and 10% AcB at 1 and 7 d under accelerated carbonation chamber (ACC) conditions.

Figure 11 presents the XRD data for samples cured under accelerated carbonation conditions (ACC) at 1 and 7 d, comparing 0% and 10% AcB. The same phases were detected in the NCC samples. A notable reduction in the portlandite phase intensity occurred from 3 d onwards, with portlandite being seemingly absent after 7 d. These results indicate complete carbonation at 7 d, which is consistent with the enhanced mechanical properties observed under ACC [35,107,128,129]. Additionally, the blue line (PM-0-1D) exhibited a slightly higher intensity than did the green line (PM-10-1D), supporting the inference that the AcB-modified sample (PM-10-1D) was more extensively carbonated than was the control sample (PM-0-1D). To date, no studies have explored the effects of accelerated carbonation on mortars incorporating olive pit-derived active biochar. Accelerated carbonation favoured the formation of CaCO_3_ from portlandite detected under NCC, even after 7 d under ACC, which increased the mechanical and physical properties of the samples.

### 4.4. Carbon Capturing

#### 4.4.1. Carbonation Depth

**Figure 12:**
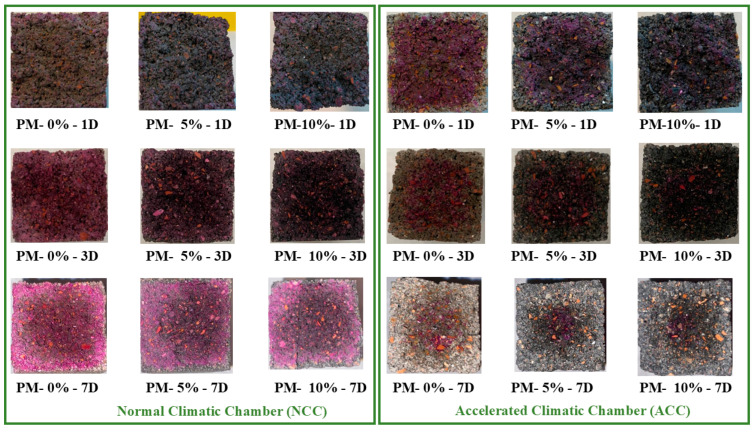
Carbonation depth using a phenolphthalein indicator at 1, 3, and 7 d of curing in both curing environments.

Figure 12 and Table 4 present the carbonation depths measured after 1, 3, and 7 d of curing in both environments (using a pH indicator). Although crucial to this research, carbonation depth measurements with a pH indicator are considered the least accurate measurement technique [133] and should be viewed as qualitative, although image processing could potentially make them quantitative [134]. However, such measurements remain useful for comparison with results obtained via other methods.

In both curing environments, increasing the AcB content led to greater carbonation, indicating that the incorporation of olive stone-derived biochar promoted accelerated carbonation in the mortar mix [86]. These results may also be related to the higher water absorption observed in Figure 8, which enhances CO_2_ diffusion and thus increases carbonation [135].

As the carbonation advanced, the degree of carbonation increased, with the samples cured in ACC being fully carbonated by day 7. The chemical reactions between CO_2_ and hydration products such as portlandite form calcium carbonate. This process has been extensively studied and documented [35,86,107,110,128].

**Table 4:** Depth of carbonation of mixes (mm).

	NCC			ACC		
	0%AcB	5% AcB	10% AcB	0% AcB	5% AcB	10% AcB
1 d	0	0	0	3	3.2	3.3
3 d	0	0	0	4.1	4	4
7 d	1	1.4	2.1	8.7	9.1	9.3

#### 4.4.2. Thermogravimetric Analysis of Mortar

**Figure 13:**
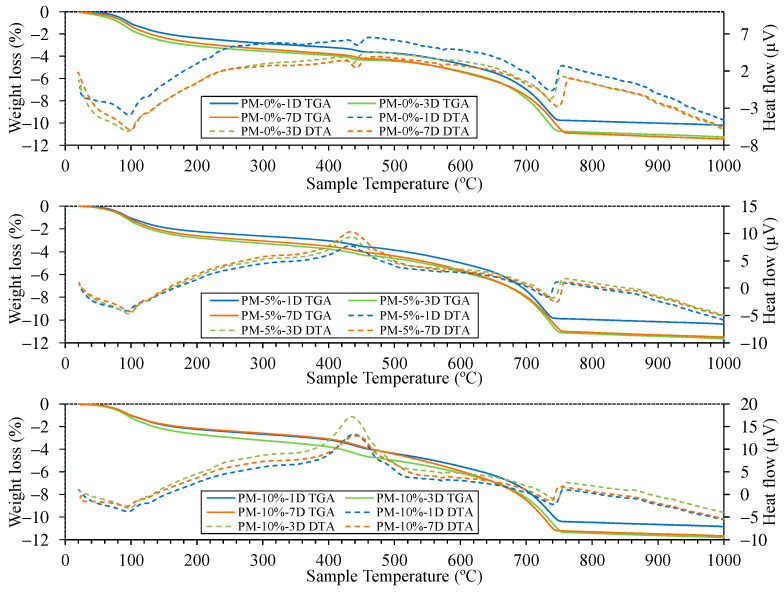
TGA/DTA of the different mixes at 1, 3, and 7 d of curing under normal climatic chamber (NCC) conditions.

**Figure 14:**
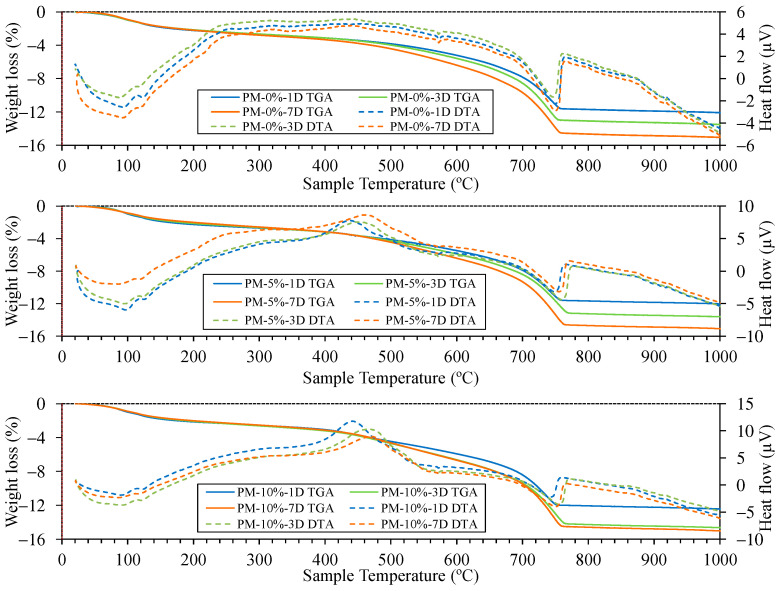
TGA/DTA of the different mixes at 1, 3, and 7 d of curing under accelerated carbonation chamber (ACC) conditions.

Figure 13 and Figure 14 show the TGA/DTA results for the mixes studied under both NCC and ACC conditions. In all cases, the TGA/DTA curves define five key regions. Notably, the onset of degradation at each stage can vary depending on factors such as the heating rate, type of cement, curing conditions, and additives used [94]. Moreover, the total mass loss increased with the percentage of AcB in both curing environments [86]. The mass losses observed between room temperature and 105 °C were attributed to the evaporation of free water. This loss could have been avoided by pre-drying all samples; however, the authors did not support this approach, as certain samples may have still retained moisture owing to the incomplete evaporation of free water [94]. The samples containing AcB exhibited slightly higher mass losses in this range owing to the higher water absorption capacity of the material, which is consistent with the absorption values shown in Figure 8.

The decomposition of C-S-H gel and ettringite, as well as the dehydration of C3A and C4AF, occurred between 105 and 400 °C [35,85,86,94]. The dehydroxylation of calcium hydroxide (portlandite) occurred between 400 and 480 °C [82,107,110,113,136], and this was identified by the endothermic peak visible on the DTA curve. This peak was observed at all curing ages in the NCC samples without AcB (control mix). However, under ACC conditions, this peak was almost entirely absent, indicating portlandite carbonation [84,85,86]. In the samples containing AcB (particularly those with 5% and 10% AcB), the portlandite peak under NCC appeared to be ‘masked’. The reaction transitioned from endothermic to exothermic. This shift is attributed to an exothermic peak appearing between 440 and 460 °C. This peak was caused by the calcination of organic material in AcB [81,116,137]. Interestingly, this AcB calcination peak was delayed under the ACC conditions, occurring between 460 and 480 °C. This delay indicates that AcB reacted with CO_2_, as was also observed by Maljaee et al. [94].

The decarbonation of well-crystallised calcite, formed by the carbonation of portlandite, occurred between 550 and 1000 °C [94]. In this study, the total calcium carbonate (CC) content was calculated based on the decarbonation region (550 to 1000 °C). To exclude the weight loss from AcB within the decarbonation range [84,86], we focused solely on the carbonation effect (comparing NCC to ACC) for (i) samples containing the same percentage of AcB and (ii) the same curing age (Table 5). Several studies adopted similar methods [85,94]. The percentage of CO_2_ uptake was introduced to define the degree of carbonation in the cement mortar and was calculated using the following equations (Equations (1) and (2)):

(1) $${CaCO}_{3}=\left(m_{1000}-m_{550}\right)$$

(2) $${CO}_{2}uptake\left(\%\right)={{CaCO}_{3}}_{ACC}-{{CaCO}_{3}}_{NCC}$$

It is important to highlight that accelerated carbonation acted as a CO_2_ sink in all studied samples when comparing the NCC and ACC conditions. These phenomena have been previously demonstrated by Suescum-Morales et al. [35] in mixes with characteristics similar to those analysed in this study. Under accelerated carbonation, the addition of 5% and 10% AcB to the samples significantly enhanced CO_2_ uptake, increasing by 37% and 88%, respectively, after 1 d, compared with the reference sample (PM-0%-1D with 2.44% versus PM-5%-1D with 3.38% and PM-10%-1D with 4.64%). At 3 d, CO_2_ uptake improved by 51% and 85% with the addition of 5% and 10% AcB, respectively. The best results were observed after 7 d, with an 87% improvement for the 5% AcB sample and a remarkable 147% increase for the 10% AcB sample.

However, no comparable studies have used AcB as an additive in porous mortars cured via accelerated carbonation while performing such calculations. The closest reference is the study of M. Xu et al. [84], which used 7% biochar and reported a 33% improvement in CO_2_ capture capacity. The TGA/DTA analysis demonstrated that AcB could effectively function as a CO_2_ capture additive in porous cement-based materials, as it enhanced the capture capacity by up to 147% under accelerated carbonation conditions.

#### 4.4.3. Ultra-Fast Test Method for CO_2_ Capture Capacity Under High Pressure

**Figure 15:**
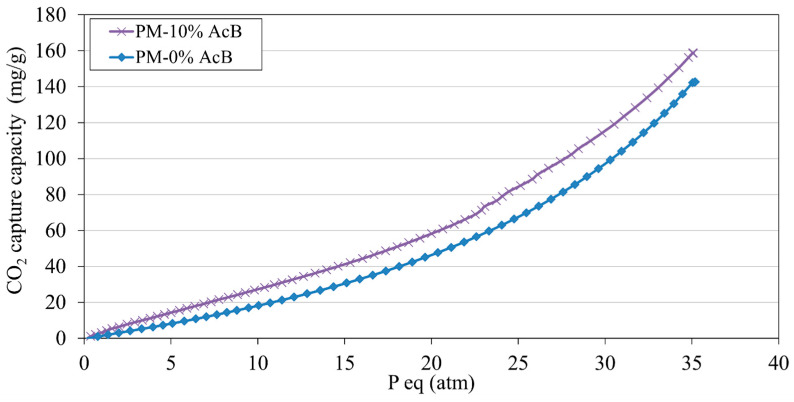
CO_2_ capture capacity (isotherms) for PM-0% and PM-10%.

The CO_2_ capture capacities at pressures ranging from 0 to 35 atm were analysed for the PM-0% and PM-10% samples (Figure 15), using the mould shown in Figure 1 [138]. The specimens were evaluated 24 h post-production; however, they were placed in a sample holder 2 h after manufacturing. For PM-0% (reference), the capture capacity increased proportionally with the rise in pressure, reaching a maximum at 35 atm. The maximum capture capacity was recorded at 142.69 mg/g.

The effect of incorporating 10% AcB on the CO_2_ capture capacity was also examined, as shown in Figure 15, using isotherms for the PM-10% sample. As with the reference, the capture capacity increased with increasing pressure, peaking at 35 atm. At this pressure, the maximum capture capacity was recorded at 158.81 mg/g. This improved capture capacity, as suggested by the isotherm data, indicates that CO_2_ pressure is a key variable influencing the effectiveness of AcB as a CO_2_ capture additive. A more comprehensive investigation of these factors should be conducted in future studies. The significance of this finding is evident; no prior research has explored the CO_2_ capture capacity of porous mortars incorporating active carbon from olive stones under high-pressure conditions.

No comparable references were available for similar mixtures, making these data unique. The ultrafast method employed here offers several advantages for calculating the CO_2_ capture capacity, including: (i) rapid results, with testing completed within 6–8 h; (ii) the elimination of the need to fabricate two 4 × 4 × 16 specimens (one for reference and one for analysis); (iii) no requirement to grind both samples for TGA/DTA analysis; (iv) removal of the need for two separate TGA/DTA tests; and (v) the omission of comparative calculation steps.

## 5. Conclusions

This study investigated the potential use of activated biochar derived from olive stone waste and recycled masonry aggregates (RMA) in the production of porous mortars. The main focus was to assess how different proportions of biochar (0%, 5%, and 10%) impact the mechanical properties, CO_2_ capture capacity, and carbonation behaviours of these materials under normal and accelerated carbonation curing conditions. The findings demonstrate that incorporating biochar not only enhances the strength and durability of mortars but also significantly boosts their CO_2_ capture potential, which provides a sustainable solution for the construction industry.

- The initial setting time (300 min) of the cement samples containing 0.5%, 1%, 3%, 15%, and 20% activated biochar (AcB) did not vary compared to the reference sample (0% AcB), confirming the compatibility of AcB with cement-based materials.
- The incorporation of AcB derived from olive stone waste into porous mortars, along with recycled masonry aggregates (RMA), significantly enhanced the mechanical performance of the materials. Specifically, the addition of 10% biochar increased the compressive strength by 110%, 105%, and 106% after 1, 3, and 7 d of curing, respectively. The flexural strength followed this trend, with higher values observed particularly during the early curing stages.
- Compressive strength due to accelerated carbonation increased with AcB content. The samples containing 10% AcB achieved a compressive strength of 11.32 MPa at 3 days under accelerated carbonation curing, surpassing the 11.29 MPa value at 7 days under normal curing conditions. This highlights the potential of activated biochar in combination with accelerated carbonation, promoting microstructural densification through CaCO_3_ formation and pore filling.
- The incorporation of activated biochar (AcB) into mortars increased dry bulk density and reduced water-accessible porosity, enhancing mechanical properties due to the pore-filling effect. However, water absorption exhibited an increasing trend with higher AcB content, attributed to its higher fine particle content and larger specific surface area.
- The inclusion of AcB enhanced CO_2_ sequestration efficiency, increasing carbonate precipitation, which resulted in a 147% increase in CO_2_ capture in the studied mix containing 10% AcB.

This study presents a sustainable solution for the construction industry by utilising recycled masonry aggregates and biochar from olive stone waste, enhancing mortar strength and CO_2_ capture capacity, thereby contributing to carbon footprint reduction. Its application in precast concrete, combined with accelerated carbonation curing, optimises strength in a shorter time while reducing costs and energy consumption. However, further research is required to assess its long-term durability and evaluate its scalability in industrial applications to maximise its impact on sustainable construction.

## Figures and Tables

**Table 1:** Physical properties of masonry aggregate (RMA).

Material	Dry Bulk Density g/cm^3^	Water Absorption %	Sand Equivalent %
RMA	2.14	9.00	0.2

**Table 2:** Dosage of the mixes studied (per cubic metre).

	PM-0 (kg/m^3^)	PM-5 (kg/m^3^)	PM-10 (kg/m^3^)
Fraction > 4	-	-	-
Fraction 2/4	241.1	241.1	241.1
Fraction 1/2	534.6	534.6	534.6
Fraction 0.5/1	658.1	658.1	658.1
Fraction 0.25/0.5	-	-	-
Fraction 0.125/0.25	174.7	174.7	174.7
Fraction < 0.125	-	-	-
CEM II/A-L 42.5R	481.7	481.7	481.7
Activated Carbon	0	14.4	28.8
Absorption water *	144.8	144.8	144.8
Effective water	192.7	192.7	192.7
*w*/*c* **	0.3	0.3	0.3

* Is the aggregate absorption water calculated according to Table 1. ** Is the effective water/cement ratio.

**Table 3:** Chemical composition of RMA, cement, and AcB obtained from XRF (%).

Oxides	RMA	Cement	AcB
F_2_O	0.74	-	-
Na_2_O	0.71	0.36	0.04
MgO	1.65	1.42	0.24
Al_2_O_3_	10.79	3.19	0.05
SiO_2_	34.44	13.48	0.18
P_2_O_5_	0.12	0.07	0.16
SO_3_	2.52	3.17	0.17
Cl^-^	0.05	0.05	0.20
K_2_O	2.18	0.90	0.71
CaO	12.18	51.49	1.20
TiO_2_	0.54	0.12	-
Cr_2_O_3_	0.02	-	-
MnO_2_	0.06	0.08	-
Fe_2_O_3_	3.55	2.10	0.06
NiO	-	0.02	-
ZnO	0.02	-	-
SrO	0.03	0.05	-
BaO	0.03	-	-
Balance CO_2_	30.61	23.50	96.98
Total	100.00	100.00	100.00

**Table 5:** Estimated CO_2_ uptake and quantification of CaCO_3_ formed in mixes.

Age	Mix	CaCO_3_	CO_2_ Uptake	CO_2_ Uptake
		(%)	(%)	(g/t)
	PM-0%–1D-NCC	6.66	2.44	24,662.00
1 Day	PM-0%-1D-ACC	9.13		
	PM-5%-1D-NCC	6.79	3.38	33,783.31
	PM-5%-1D-ACC	10.16		
	PM-10%-1D-NCC	6.85	4.64	46,432.11
	PM-10%-1D-ACC	11.49		
	PM-0%-3D-NCC	7.04	2.56	25,564.77
3 Days	PM-0%-3D-ACC	9.59		
	PM-5%-3D-NCC	7.38	3.86	38,575.77
	PM-5%-3D-ACC	11.24		
	PM-10%-3D-NCC	7.19	4.74	47,445.87
	PM-10%-3D-ACC	11.93		
7 Days	PM-0%-7D-NCC	7.31	2.88	28,797.82
	PM-0%-7D-ACC	10.19		
	PM-5%-7D-NCC	7.47	5.37	53,743.46
	PM-5%-7D-ACC	12.85		
	PM-10%-7D-NCC	7.73	7.10	71,026.66
	PM-10%-7D-ACC	14.83		

## Data Availability

The original contributions presented in this study are included in the article. Further inquiries can be directed to the corresponding authors.
